# Nutritional Education Needs and Preferences of Sports Volunteers: Access, Expectations, and Forms of Support

**DOI:** 10.3390/nu16203568

**Published:** 2024-10-21

**Authors:** Mateusz Rozmiarek

**Affiliations:** Department of Sports Tourism, Faculty of Physical Culture Sciences, Poznan University of Physical Education, 61-871 Poznan, Poland; rozmiarek@awf.poznan.pl

**Keywords:** volunteering, sport, nutrition, volunteer management, sports event management, dietary preferences, diet, nutritional education, qualitative research, interviews

## Abstract

The aim of this study was to analyze the needs and preferences of sports volunteers regarding nutritional education, with particular emphasis on the availability of educational materials and expectations towards event organizers. The methodology was grounded in a qualitative approach, employing detailed individual interviews (IDIs) with seventeen volunteers (n = 17) who were actively involved in various sporting events, including races, triathlons, and athletic competitions at local, national, and international levels. This sample size was justified as it was sufficient to achieve data saturation, meaning no new significant themes emerged after these interviews. The results indicate that most participants feel a lack of access to reliable information about nutrition, with 70% (n = 12) indicating a need for educational materials, which limits their ability to make informed dietary decisions. Volunteers expect event organizers to provide educational materials and prefer a variety of practical forms of education, such as interactive workshops and accessible online resources. While the volunteers expressed a desire for improved nutritional education, further investigation is needed to establish a direct link between this education and potential enhancements in their performance and well-being. For this reason, greater attention should be paid to the nutritional education of volunteers, which is a key element of their preparation to work in high-stress and physically intense conditions.

## 1. Introduction

Sports volunteering is an integral part of organizing sporting events, both at the local and international levels [1,2,3]. Volunteers support various aspects of event organization, ranging from logistics to athlete care and technical support [4,5]. Their role in the functioning of such events is invaluable, and their presence often determines the logistical success of the entire undertaking. Volunteering in sports activities typically occurs during two main situations: the duration of championships and training or preparation sessions. The nature of the volunteer work can vary significantly depending on the duration and intensity of the event, the specific demands of the sport, and whether the activities take place indoors or outdoors. Nevertheless, despite the crucial contribution of volunteers, their specific needs, especially those related to nutrition, remain largely neglected. Given the increasing demands associated with the intensity of work, long hours, and the activities undertaken, it is worth considering how proper nutrition could influence the performance and overall condition of volunteers.

From the perspective of the scientific literature, the area of athlete nutrition is well-researched [6,7,8,9]. Studies on the physiology of physical exertion clearly indicate that a properly balanced diet is a key factor influencing the recovery capabilities, muscle strength, and mental resilience of athletes [10]. Undeniably, what an athlete consumes directly affects their ability to maintain a high level of performance during competitions [11,12,13]. However, despite certain similarities between the intensity of sports volunteers’ work and the physical challenges faced by athletes, the nutritional needs of the former have not received similar attention in research and contain fundamental differences. Understanding the specific nutritional needs of sports volunteers is crucial for their performance during sporting events. The work of a volunteer, often performed under significant physical and mental stress, requires maintaining stable energy levels and adequate recovery [14]. Unlike athletes, who benefit from the care of dietitians and have access to carefully designed nutritional plans, volunteers do not need to present maximized fitness levels but only an individual level of fitness sufficient to perform their tasks. A diet that is too poor or inappropriate can contribute to decreased performance and even weaken the body’s immune system, which in the long run may lead to fatigue or exhaustion [15].

One of the key issues faced by volunteers is the limited access to reliable information about nutrition [16]. Although modern technologies offer broad access to content related to a healthy lifestyle, the quality of this information can be questionable [17,18]. Many blogs, websites, and social media profiles promote content that may be fragmented, unverified, or based on trendy yet not always scientifically supported nutritional theories [19,20]. For volunteers who lack specialized knowledge, this can lead to nutritional decisions that do not support their optimal condition and health.

An important area to explore in the context of sports volunteers is their awareness of how diet affects both physical and mental health. Although the topic of healthy eating is increasingly present in public debate [21,22], not everyone has access to reliable educational materials, especially when they are delivered in a complicated or impractical manner. For volunteers who operate under stress and time pressure, such as those involved in sporting events, the nutritional choices they make can be significantly compromised. Inadequate nutrition can lead to decreased performance, fatigue, and impaired mental health, which in turn can affect their overall satisfaction and motivation. Therefore, nutrition education should be tailored to their specific needs in order to realistically improve their performance and well-being during these high-pressure situations. This is particularly important given that previous research on volunteer satisfaction and motivation [23,24,25,26,27,28,29,30], which has been the most extensively studied element of their functioning, has not explored this aspect. Addressing the challenges posed by inadequate nutrition education is urgent, as reliable and accessible resources could make a significant difference in the effectiveness of sports volunteers.

The role of event organizers in supporting volunteers should, therefore, also include issues related to their nutrition. Organizers, who are responsible for ensuring the working and resting conditions for volunteers, have the opportunity to provide them with educational materials about healthy eating, which would help volunteers better understand how diet affects their performance and how they should plan their meals before, during, and after sporting events. Many volunteers already possess some level of nutritional knowledge due to their involvement in physical activities; however, this knowledge can vary widely among individuals. The introduction of such support could improve not only their well-being but also their engagement and effectiveness during work.

Preferences regarding forms of nutrition education may vary depending on various factors such as age, level of education, experience in volunteering, and availability of technology. While it is true that many volunteers are likely to have some background in nutrition, particularly those actively participating in sports, there is still a notable gap in tailored educational resources that address the specific needs of volunteers across different sports and roles. Younger volunteers may prefer modern solutions, such as mobile applications or interactive online courses, while older individuals may prefer more traditional forms, such as printed materials or direct workshops. Importantly, access to nutrition education and materials supporting healthy eating may also be limited by various barriers. Not only can a lack of time or an overload of responsibilities on the part of event organizers be problematic, but financial barriers, technological limitations, or a lack of awareness among organizers can also contribute to a low level of nutritional knowledge among volunteers. These barriers highlight the need for structured programs that can systematically address these gaps, ensuring all volunteers, regardless of their background, receive adequate nutrition education.

The objective of this study is to determine the needs and preferences of sports volunteers regarding nutrition education, with a particular focus on the availability of educational materials, expectations of event organizers, and preferred forms of support. Specifically, this study examined the availability of educational materials (e.g., information on the quality of meals, their energy value, the role of hydration, and meal timing), methods of knowledge delivery (e.g., workshops, online resources, printed materials), and the relevance of the information provided in the context of the volunteers’ specific roles and activities. To better understand these issues, two research hypotheses were formulated:

**Hypothesis** **1.** *Volunteers expect that event organizers will provide access to educational materials on healthy eating*.

**Hypothesis** **2.** *Sports volunteers prefer diverse forms of nutrition education*.

This study aims to investigate these issues through in-depth interviews with sports volunteers to better understand their experiences, needs, and preferences regarding nutrition education.

## 2. Materials and Methods

The research methodology is based on a qualitative approach utilizing in-depth interviews (IDIs) [31]. The IDI is considered one of the most effective methods for gaining profound insights into individuals and examining various topics [32]. Thanks to the individual approach to respondents, as opposed to methods such as focus groups, it allows for more detailed results and greater engagement in discussions on sensitive or personal topics [33].

### 2.1. Sample Selection

This study involved 17 sports volunteers, including nine women (designated as V3-4, V7, V10, V12-14, V16-17) and eight men (V1-2, V5-6, V8-9, V11, V15). The volunteers participated in the organization of different sporting events, such as races of different distances, triathlons, and athletic competitions. As part of the study, interviews were conducted regarding the volunteers’ expectations for access to educational materials on healthy eating as well as their preferences regarding forms of support. The aliases of the participants, along with their ages, are presented in Table 1. All participants were of legal age and had experience working at various sports events organized in the past two years. The sample was selected through purposive sampling, meaning that participants were chosen based on specific criteria. The key requirements were: (1) active participation in sports volunteering for at least two years—ongoing collaboration with organizations responsible for volunteer management at sporting events, ensuring the continuous presence of volunteers at sporting events; (2) experience gained at sports events at the local, national, and international levels. Participants who were not of legal age and those who did not meet the required criteria outlined in the key requirements were excluded from the study. Participants held diverse roles in volunteering, which allowed for the collection of varied perspectives on the dietary requirements and habits related to their roles.

### 2.2. Justification for Sample Size

The sample size was defined according to qualitative research principles, which dictate that the number of participants is typically established based on the point of data saturation. Achieving saturation means that subsequent interviews do not yield new significant themes or information that could meaningfully affect the comprehension of the studied topic. In the case of this research, data saturation was achieved after conducting seventeen interviews, demonstrating that the sample was adequate for obtaining complete and thorough information. Such a sample size is also supported in the literature on qualitative research methodology [34,35].

### 2.3. Procedure

The investigation was performed through semi-structured interviews, allowing participants to freely express their thoughts in open-ended questions. This methodology enabled more detailed responses and the discovery of unexpected themes regarding the needs and preferences of sports volunteers in the field of nutrition education. The interviews focused on three key areas: volunteers’ expectations of event organizers concerning access to educational materials, their preferences for different forms of support, and the barriers associated with the availability of nutrition education. This approach facilitated an understanding of the volunteers’ attitudes toward learning about nutrition, taking into account their individual needs.

Conversations with participants took place from 1 July to 15 August 2023. Each interview lasted approximately 90 min and addressed various issues related to nutrition within the realm of sports volunteering, with an emphasis on the availability of educational materials and preferred forms of support. The interviews were captured with the participants’ permission.

The entire research procedure was in accordance with ethical scientific standards. Participants were fully briefed on the study’s goals, its nature, and the voluntary aspect of their involvement. Each respondent gave informed consent to take part in the study and for the recording of the interviews. Anonymity and confidentiality were ensured, and the collected data were presented in a manner that prevented the identification of participants.

### 2.4. Data Analysis

The information gathered from the interviews underwent analysis following a four-step thematic analysis process: (1) Transcribing the recordings and preliminary familiarization with the collected material; (2) classifying participants’ responses and identifying common themes; (3) organizing the classifications into wider thematic categories; (4) analyzing the results and deriving conclusions from the recognized themes. Each of these stages was conducted following the established guidelines for the rigor of qualitative research, ensuring high reliability and adequacy of the obtained conclusions.

To increase the transparency and thoroughness of the analysis, a multi-phase and iterative approach to thematic analysis was adopted. Following the initial transcription of the interviews, the transcripts were reviewed multiple times to thoroughly grasp the participants’ statements. Data coding occurred at two levels: (1) Open coding, which revealed potentially significant themes, and (2) axial coding, which clustered thematic connections and enabled the recognition of key motifs. This process was facilitated by analytical tools like NVivo (version number 12), which guaranteed a systematic coding approach.

All codes were subsequently compared and organized to refine the thematic categories. The analysis results were compared with the scarce existing research in related thematic fields, enabling the conclusions to be placed within a wider context. The author used a meticulous method for coding and classifying the data. To decrease subjectivity and increase the objectivity of the results, the researcher frequently reassessed the identified motifs and compared them with the original codes to ensure consistent interpretation.

## 3. Results

Below are the key findings that emerged from discussions, supported by quotes from the participants.

### 3.1. Expectations of Volunteers Regarding Access to Educational Materials on Healthy Eating

In the conducted study, the vast majority (n = 12) indicated a lack of access to educational materials on healthy eating during organized events. V2 stated, “We didn’t have any materials; it wasn’t even mentioned”. Similar feelings were echoed by V3, who noted, “The organizers didn’t provide us with any information or resources on nutrition. Do I think they should? I think it wouldn’t hurt”. V8 also pointed out this issue, saying, “I don’t recall any educational materials. However, I think they could be useful”.

Other volunteers confirmed these observations, emphasizing that the lack of access to information limited their abilities. V12 stated, “One time, one of the women felt unwell. I thought maybe she was low on sugar and suggested she eat a candy bar I had with me, but I really don’t know if that was the right thing to do or if I harmed her”. V5 shared similar sentiments, saying, “In my opinion, the organizers should at least inform us on a basic level about the role of nutrition so that we have at least a fundamental understanding of it”. V6 added, “I understand that these are sporting events and not directly related to nutrition, but the organizers didn’t show any interest at all, and they should have”. Another volunteer, V16, emphasized, “I didn’t receive any information. If I had materials, my knowledge would certainly be greater”. V4 stated, “Generally, during the volunteering, I don’t pay much attention to this and just do my job, but having any access to additional knowledge would always be valuable”. V10 added, “I have never had any meetings during sporting events where healthy eating was discussed. And there are always many briefings with volunteers before the event, so there was room for that”. V9 presented a similar position: “We meet so many times and share our responsibilities, but to hold some training or provide us with printed information on nutrition education, that never happened”. V15 emphasized categorically: “We never received any information. I feel that the organizers do not realize how important this is”. V17 agreed, saying, “I think the organizers do not understand how important educational materials on various topics are for us, not only in terms of nutrition but also comprehensive knowledge about the sports that are organized during the events we support. Without this, we cannot operate effectively”.

Several volunteers (n = 4) shared their experiences related to limited access to materials. V13 said, “Having participated in volunteering for several years, I have heard something about healthy eating a few times, but it was only during meetings and not in the form of materials”. V7 added, “I know it could be done better. We always said we wanted more information, but we never received a response”. V11 noted, “There were a few cases where we had access to such materials, but it was definitely not regular”. V14 criticized the regular lack of education, saying, “Discussions on this topic are definitely irregular and thus insufficient”.

Finally, one of the volunteers (n = 1), V1, had slightly different experiences. He said, “I had the opportunity to use educational materials a few times, and I was very satisfied with that. It increased my awareness of nutrition and expanded my knowledge, which is useful in everyday life”. His positive experience is an exception that seems to highlight the need for greater availability and regularity in providing materials on healthy eating by event organizers. In light of the collected opinions, it can be concluded that the research hypothesis is confirmed, and volunteers expect more care from the organizers to ensure the appropriate educational resources.

### 3.2. Preferences for Forms of Nutritional Education

In the conducted study, opinions were gathered regarding their preferences for forms of nutrition education. Two individuals (n = 2) emphasized that it is crucial for them that educational materials are easily accessible and practical. V2 stated, “I value forms of education that I can have on hand at all times. The easier the materials are to access, the better I can use them”. A similar opinion was expressed by V14, who said, “I’m interested in short instructional videos that I can watch in my free time. Such materials are the most practical for me”. Another group of volunteers (n = 3) pointed out the need for interactive and engaging forms of education. V3 said, “I like workshops where I can learn something new and immediately try it out. Theoretical approaches don’t work for me”. V4 emphasized, “Interactive sessions with experts make sense. When I can ask questions and discuss, I absorb knowledge much better”. V6 agreed, stating, “Practical group activities would probably be the best. We learn from each other, which increases our motivation”.

Further volunteers (n = 4) highlighted the importance of available online resources. V7 stated, “I appreciate the ability to use mobile apps that offer recipes and nutrition advice. It’s very convenient. Of course, the organizers of sporting events don’t promote them, but I think that’s a mistake. I try to use the Fitatu app, although I’m probably doing it incorrectly since I’ve never been shown how to use it”. V12 added, “Webinars are a great learning format for me. I can participate from anywhere, which is nice, and it makes me feel like I’m expanding my knowledge”. V17 noted, “The availability of materials in PDF format that I can download and review at my convenience would be pleasant”. This group also included V15, who added, “Practical guides in PDF format or even infographics would make sense”.

Some volunteers (n = 3) also emphasized that nutrition education should be tailored to their specific needs. V16 said, “I would like nutrition education to be more personalized. I often feel that general advice doesn’t fit everyone”. V9 added, “Knowledge about appropriate meals before and after physical activity would be very useful for preparing adequately for tasks. When I know I’ll be working physically, I will certainly prepare myself better with that knowledge”. In a similar tone, V13 noted, “It would be good to have access to materials that take different activities into account. Not everyone has the same nutritional needs”.

Among the volunteers, there were also individuals (n = 3) who expressed a desire to utilize traditional forms of education. V11 stated, “I believe that well-organized seminars or classes in classrooms are valuable. I just want to hear information from an expert to be sure that it is reliable”. V8 added, “Some of the more formal education methods can also be effective, especially when the topic is comprehensive”. V10 noted, “Maybe it would be worth combining traditional methods with modern ones to reach a broader audience”.

The last two individuals (n = 2) in the group of volunteers highlighted additional aspects of nutrition education. V5 said, “Sometimes I lack materials that are tailored to current nutrition trends. I would like to know what is currently in vogue”. V1 added, “It is important to me that education is dynamic and changes with new research. I’m curious how new information can influence nutritional decisions”.

## 4. Discussion

This study was conducted based on the methodology of in-depth individual interviews. The choice of this method stems from the desire to understand not only the basic behaviors of sports volunteers in the context of their nutritional choices but also their expectations regarding nutrition education. The method enabled a detailed examination of the needs, preferences, and obstacles that volunteers encounter concerning nutrition education issues. The qualitative approach enabled the questions to be adapted flexibly to the unique experiences of the respondents, capturing subtle differences in expectations regarding nutrition education.

The results of the conducted study reveal a serious problem regarding the lack of access to educational materials on healthy nutrition among sports volunteers. The vast majority of participants indicated that event organizers did not provide them with appropriate informational resources. A significant aspect is that a lack of knowledge about healthy eating can not only affect the well-being of volunteers but also impact the effectiveness of their actions during events. According to previous research conducted by Rozmiarek [16], the majority of sports volunteers do not focus on their diet, leading to random food choices that may negatively affect their performance. The study results also indicate the necessity of nutrition education among sports volunteers and promoting the utilization of trustworthy sources of information regarding healthy eating.

In light of these findings, event organizers could enhance nutrition education through several specific strategies. One potential approach is forming partnerships with certified nutritionists to develop tailored educational materials or hold workshops focused on healthy eating habits for volunteers. Additionally, event organizers could create online resources, such as interactive platforms or mobile applications, to provide volunteers with easy access to up-to-date nutritional guidelines. These strategies could facilitate continuous learning, providing volunteers with the necessary knowledge and tools to make informed dietary choices before, during, and after sports events.

It should be noted that the lack of access to educational materials regarding nutrition can result in decisions being made based on insufficient or inaccurate information. Previous studies highlight that improper dietary decisions can result not only in poor well-being but also in an increased risk of injuries and health problems [36,37,38,39,40]. Organizers should be aware that nutrition education is a key element not only for volunteers but also for event participants, who may be exposed to various health situations. Having the right information about nutrition can influence the overall safety and comfort of all individuals involved in the event. Therefore, it is worth considering the introduction of training and workshops that present the basic principles of healthy eating in an accessible way to enhance the competencies of volunteers and event participants in this field.

Volunteer preferences regarding forms of education are also crucial for understanding their expectations of organizers. The study results indicate the need for diverse and interactive forms of teaching, reflecting the changing approach to adult education. Contemporary research shows that active teaching methods, such as workshops or discussion sessions, are more effective compared to traditional lectures [41,42]. Therefore, event organizers should invest in educational forms that not only engage participants but also facilitate better knowledge absorption. Offering practical experiences where volunteers can acquire new skills and knowledge interactively will contribute to increasing their motivation and involvement.

The issue of personalizing nutrition education is another key aspect that requires attention. The study results indicate the need to tailor educational materials to the specific needs of volunteers, which can be especially crucial given the variety of events they participate in. Personalizing nutrition education can contribute to increasing its effectiveness and participant satisfaction, as each form of activity has its own unique nutritional requirements. Event organizers should consider collaborating with nutrition experts to develop materials tailored to different disciplines, allowing for more precise and effective preparation of volunteers for their roles.

It is also essential to consider how the Polish cultural context may influence the applicability of these findings to other countries or settings. Poland’s specific dietary customs, public health priorities, and volunteer management practices may shape volunteers’ expectations and behaviors in ways that differ from those in other regions. For example, the availability of local nutritional education resources and cultural attitudes toward healthy eating may vary, potentially limiting the direct transferability of the results. Future studies should address these cultural differences to provide insights that are more universally applicable.

The final aspect to emphasize is the importance of updating educational materials in the context of the dynamic development of knowledge about nutrition. Research shows that as new discoveries and dietary trends emerge, it is crucial for educational materials to be continuously adjusted to current recommendations and standards [43]. Volunteers highlighted that they want to stay informed about the latest information, which is especially important in light of changes in nutrition science. To effectively support volunteers, event organizers should implement mechanisms for monitoring and updating materials, ensuring that they provide knowledge that is not only current but also relevant to their needs and expectations.

## 5. Strengths and Limitations

A strength of the conducted study was the use of individual interviews, which allowed for a detailed examination of the individual experiences and preferences of volunteers regarding nutrition education. This approach provided rich, multifaceted data that facilitated an understanding of expectations concerning access to educational materials and support forms in the context of their engagement in sports volunteering. The interviews also enabled an analysis of participants’ nutritional awareness, allowing for the identification of differences in the perception of nutrition education and the obstacles they encounter in accessing information and materials on the subject.

However, this study has specific limitations that could influence the ability to generalize the findings. Firstly, the research was carried out in Poland, which may limit the relevance of the findings to a wider population of sports volunteers in other countries. Poland’s cultural context, including local dietary preferences, health education systems, and attitudes toward volunteering, may affect the applicability of the results to other regions. Differences in access to nutrition education resources across countries should be considered when interpreting the study’s findings. To gain a deeper understanding of the influence of various factors, such as cultural variations and levels of education, additional research involving a larger and more diverse participant group is essential.

Furthermore, the use of interviews might introduce subjectivity into the responses, especially regarding the assessment of one’s own educational preferences and needs. This subjectivity could potentially limit the reliability of the findings, as respondents may not always have a clear or accurate understanding of their needs. In the future, it would be worthwhile to consider utilizing quantitative methods, such as surveys or analyses of the availability of educational materials, to gather more impartial data. Quantitative methods enable more accurate results and enable the analysis of a broader dataset, which increases the reliability of the conclusions drawn. For instance, future research could aim to measure the nutritional knowledge of a larger and more diverse group of volunteers through surveys, assessing the effectiveness of existing educational resources across different sports and cultural contexts.

Such an approach can also help identify trends and correlations that could be difficult to discern in qualitative research. The introduction of these methods could greatly improve the quality of future research on nutrition education among sports volunteers and provide more relevant and universal conclusions. A possible research question for future studies could focus on the relationship between volunteers’ nutritional knowledge and their performance at events, exploring how enhanced education impacts their effectiveness and well-being.

Additionally, it is important to consider the feasibility of incorporating nutrition education into the structure of sporting events, as organizers may face budget or time constraints. While the recommendation to include nutrition education is valid, these practical limitations might affect its implementation. To address this, event organizers could explore cost-effective solutions, such as incorporating brief digital resources or collaborating with external sponsors or nutrition experts, which would allow for the dissemination of educational content without significantly increasing the logistical or financial burden. This approach would help ensure that nutrition education can be integrated into sporting events in a realistic and sustainable manner.

## 6. Conclusions

The results of the conducted study clearly indicate a significant gap in access to nutrition education for sports volunteers, which has a substantial impact on their performance and overall well-being during sports events. Although volunteers recognize the need to expand their knowledge about healthy eating, event organizers rarely provide them with appropriate educational materials. By enhancing nutrition education through various forms of support, such as interactive workshops or access to practical online resources, not only can volunteer engagement be increased, but it can also contribute to their better physical and mental condition.

To make a meaningful impact, event organizers could implement specific programs, such as partnering with certified nutritionists to conduct regular workshops focused on healthy eating habits or creating comprehensive online platforms where volunteers can access tailored nutritional resources. Successful examples include initiatives that provide meal plans and nutrition guides tailored to different types of events, ensuring that volunteers are well-informed about their dietary needs. A key step toward improving the working conditions for volunteers should, therefore, be the incorporation of nutritional aspects into the organization of sports events, which will allow for better utilization of their capabilities and aid in the overall success of the initiatives.

Improving volunteers’ nutrition education can directly influence their energy levels, focus, and stamina during events, which are critical factors in ensuring their efficiency and ability to perform assigned tasks effectively. Well-nourished volunteers are less likely to experience fatigue, dehydration, or lack of concentration, which in turn reduces the likelihood of mistakes or underperformance. As a result, their enhanced physical and mental condition can improve the overall coordination and flow of the event, contributing to its success by minimizing disruptions, ensuring smooth operations, and fostering a positive experience for both participants and organizers. Additionally, well-informed volunteers can act as ambassadors for healthy living, further reinforcing the event’s reputation and its alignment with promoting wellness.

## Figures and Tables

**Table 1:** Table of participants.

Male Volunteers		Female Volunteers	
Alias	Age	Alias	Age
V1	25	V3	20
V2	19	V4	18
V5	22	V7	18
V6	20	V10	23
V8	24	V12	19
V9	21	V13	25
V11	28	V14	18
V15	23	V16	19
		V17	20

## Data Availability

The original contributions presented in the study are included in the article; further inquiries can be directed to the corresponding author.
